# Differentially Detectable Mycobacterium tuberculosis Cells in Sputum from Treatment-Naive Subjects in Haiti and Their Proportionate Increase after Initiation of Treatment

**DOI:** 10.1128/mBio.02192-18

**Published:** 2018-11-20

**Authors:** Kathrine McAulay, Kohta Saito, Thulasi Warrier, Kathleen Frances Walsh, Laurent Daniel Mathurin, Gertrude Royal-Mardi, Myung Hee Lee, Oksana Ocheretina, Jean William Pape, Daniel W. Fitzgerald, Carl F. Nathan

**Affiliations:** a Center for Global Health, Weill Cornell Medicine, New York, New York, USA; b Department of Microbiology & Immunology, Weill Cornell Medicine, New York, New York, USA; c Les Centres GHESKIO, Port-au-Prince, Haiti; Harvard School of Public Health; University of Pittsburgh School of Medicine; Harvard T. H. Chan School of Public Health

**Keywords:** *Mycobacterium tuberculosis*, differentially detectable bacteria, early bactericidal activity, rifampin, viable but nonculturable bacteria

## Abstract

Recent reports indicate that the sputum of 80% or more of treatment-naive subjects with tuberculosis recruited in England or South Africa contained more viable Mycobacterium tuberculosis cells detected by limiting dilution (LD) in liquid culture than detected as CFU. Efforts to generate such differentially detectable (DD) M. tuberculosis populations *in vitro* have been difficult to reproduce, and the LD assay is prone to artifact. Here, we applied a stringent version of the LD assay to sputum from 33 treatment-naive, HIV-negative Haitian subjects with drug-sensitive tuberculosis (TB) and to a second sputum sample after two weeks of standard treatment with isoniazid, rifampin, pyrazinamide, and ethambutol (HRZE) for 13 of these subjects. Twenty-one percent had statistically defined levels of DD M. tuberculosis in their pretreatment sputum at an average proportional excess over CFU of 3-fold. Sixty-nine percent of those who received HRZE had statistically defined levels of DD M. tuberculosis in their sputum, and of these, the mean proportionate excess over CFU was 7.9-fold. Thus, DD M. tuberculosis is detectable in pretreatment sputum from a significant proportion of subjects in the Western Hemisphere, and certain drugs or drug regimens, while reducing CFU, may at the same time increase the proportional representation of DD M. tuberculosis among the surviving bacilli. Monitoring DD M. tuberculosis may improve our ability to predict the efficacy of efforts to shorten treatment.

## Abstract (precis)

Measurement of the reduction in CFU in sputum of patients with TB up to 2 weeks after the initiation of treatment is the gateway test for a new TB treatment. Reports have suggested that CFU assays fail to detect the majority of viable M. tuberculosis cells in sputum samples from the majority of patients when the number of M. tuberculosis is estimated by limiting dilution (LD). In an effort to avoid potential methodologic confounders, we applied a modified version of the LD assay in a study of a geographically distinct population. We confirmed that differentially detectable (DD) M. tuberculosis is often found before treatment, albeit at lower proportionate levels than in earlier reports. Strikingly, the prevalence and proportionate representation of DD M. tuberculosis increased during standard treatment. Sublethal exposure to certain antibiotics may help generate DD M. tuberculosis cells or enrich their representation among the surviving bacteria, and this may contribute to the need for prolonged treatment with those agents in order to achieve durable cures.

## INTRODUCTION

The urgency of finding new treatment regimens for tuberculosis (TB) is driven by TB’s leading position as a cause of death from infectious disease and the spread of resistance to all approved TB drugs in current use (1, 2). The emergence of resistance is fostered by treatment regimens that are very long and hard to complete. Thus, the focus of much research in TB drug development is for new agents and regimens that can shorten treatment. New agents and regimens are usually required to demonstrate efficacy in an early bactericidal activity (EBA) study, lasting no more than 2 weeks, before they can progress to longer, more expensive, and more informative clinical trials. A positive effect in an EBA study consists of a predetermined level of reduction in the number of CFU or CFU equivalents per milliliter of sputum (3). CFU equivalents are often estimated from the rate of growth in liquid of a bacterial suspension prepared at a single concentration from sputum, based on the consumption of oxygen in a mycobacterial growth indicator tube (MGIT) (4).

The prolonged duration of treatment regimens and the methodology of EBA assays present two interrelated concerns. First, it is widely hypothesized that M. tuberculosis in certain metabolic states may remain viable while being temporarily unable to replicate, and in such states, it shows complete or partial phenotypic tolerance to most approved TB drugs (5–8). Second, at least two groups have reported that the majority of viable M. tuberculosis cells in the sputum of the majority of untreated subjects with pulmonary TB are not detectable as CFU (9–11) but can be detected instead by limiting dilution (LD) in liquid culture, where their concentration in the original sample can then be estimated using the most probable number (MPN) statistic (12). When the number of CFU per unit volume of sputum is subtracted from the number of M. tuberculosis cells per unit volume of sputum estimated by the LD-MPN method, the difference has been called differentially culturable TB (DCTB) (9) or differentially detectable M. tuberculosis (DD M. tuberculosis) (13). Where studied, DD M. tuberculosis appeared to decrease in number upon initiation of treatment less quickly than did CFU (10), suggesting that DD M. tuberculosis may be phenotypically tolerant to the treatment. Previous studies have suggested that culture medium conditioned by log-phase M. tuberculosis has a varied effect on the quantification of DD M. tuberculosis present in patient sputum when added to the medium used for limiting dilution (9, 10).

These considerations raise the concern that standard EBA studies may not be well suited to the identification of agents or regimens that can overcome the phenotypic tolerance of poorly replicating M. tuberculosis and thereby shorten therapy. LD-MPN assays are too cumbersome for routine clinical use; however, research based on them has the potential to build a case for seeking more practical approaches to accomplish the same goal, the ability to quantitate viable but temporarily nonreplicating M. tuberculosis populations in patient-derived specimens as a way to monitor the potential ability of agents or regimens to shorten treatment.

Skepticism has become attached to the concept of DD M. tuberculosis because various protocols for generating such populations from M. tuberculosis in mice or *in vitro* have led to few instances of independent verification by LD-MPN assays in different laboratories. Here, we applied a version of an independently replicated LD-MPN assay protocol to sputum collected from HIV-negative adults in Haiti with drug-sensitive pulmonary TB (13). Earlier reports involved subjects in England (10), some of whom were likely to have emigrated from South Asia and elsewhere, and subjects in South Africa, some of whom were HIV positive (9). We studied three subjects during method validation who received standard therapy with HRZE and 30 subjects in a clinical trial of nitazoxanide, the results of which will be reported separately. In the clinical trial, 10 subjects were randomized to serve as a control group receiving HRZE. Here, we present an analysis of DD M. tuberculosis detected using the LD-MPN assay, with and without M. tuberculosis culture filtrate, in the sputum samples from all 33 subjects before treatment and at the 2-week point in the 13 subjects who received HRZE.

## RESULTS

### Study population.

The study was approved by the institutional review boards of Weill Cornell Medical College and the GHESKIO Centers, and the nitazoxanide trial was registered with Clinicaltrials.gov (registration no. NCT02684240). All participants provided written informed consent. Subjects with no reported history of TB or its treatment or any significant comorbidity were recruited from the clinic if they had a clinical and radiographic picture consistent with pulmonary TB and a positive Xpert MTB/RIF test that did not indicate rifampin resistance. The sputum smear was positive in 82%. The 33 subjects (64% male) had a median age of 25 years. Radiologic evaluation of the lungs was recorded for 30 of the 33 subjects (62% male; median age, 34 years), of whom 67% had cavities and 57% had bilateral disease (Table 1).

**TABLE 1:** Patient demographics

Demographics	All subjects (*n* = 33^*a*^)	Subjects followed up at day 14 (*n* = 13^*a*^)
Male, *n* (%)	21 (63.6)	8 (61.5)
Age, median (IQR)	25 (22–34)	34 (24–38)
Presence of cavities,^*a*^ *n* (%)	20 (66.7)	6 (60)
Bilateral disease,^*a*^ *n* (%)	17 (56.7)	6 (60)
AFB smear positive^*b*^	27 (81.8)	12 (92.3)
+, *n* (%)	1 (3.0)	1 (7.7)
++, *n* (%)	16 (48.5)	7 (53.8)
+++, *n* (%)	10 (30.3)	4 (30.8)
GeneXpert positive^*c*^	33 (100)	13 (100)
Very low, *n* (%)	3 (9.1)	3 (23.1)
Low, *n* (%)	2 (6.1)	1 (7.7)
Medium, *n* (%)	8 (24.2)	3 (23.1)
High, *n* (%)	20 (60.6)	6 (46.2)

a Presence of cavities and bilateral disease recorded for 30 patients, 10 of whom were followed up at day 14. The respective *n*’s for columns 2 and 3 are therefore 30 and 10.

b Detection of acid-fast bacilli.

c Xpert MTB/RIF assay. High, cycle threshold (*C_T_*) <16; medium, *C_T_* 16-22; low, *C_T_* 22-28; very low, *C_T_* >28.

### Parameters for quantitation of viable M. tuberculosis in sputum.

The LD-MPN method for enumeration of viable M. tuberculosis uses replicate series of samples diluted to a point past which no bacterial growth is observed for at least two consecutive weeks after bacterial growth was observed in the more concentrated dilutions. Using sufficient numbers of independent dilution series allows for the generation of a 95% confidence interval for the estimate of number of viable bacteria in the undiluted sample. The tendency for M. tuberculosis to stick to surfaces and to clump makes the assay prone to artifacts, such as a bacillus adhering to a pipette tip past one dilution and coming off in a subsequent dilution, or a clump transferring as a single particle through one dilution and coming apart in a subsequent dilution. Previous work developed an LD-MPN protocol that minimized potential artifacts by the criterion that the LD-MPN quantification of mid-log-phase M. tuberculosis predicted a number of viable bacteria that was indistinguishable from the number determined as CFU (13). For example, the protocol calls for changing pipette tips at each dilution, because failure to do so sometimes led to a >10,000-fold artifactual increase in the LD-MPN estimate over CFU enumeration for mid-log-phase M. tuberculosis. Additional steps in the protocol included using the same rather than separate dilution series for CFU and LD, using filtered pipette tips, and mixing extensively within the pipette tip before and after transferring each aliquot in a dilution series. An independent lab recapitulated both the apparent lack of DD M. tuberculosis in an M. tuberculosis population in log-phase growth *in vitro* and the emergence of a DD M. tuberculosis population in M. tuberculosis that was first starved and then exposed to rifampin *in vitro* (13). In the present work, this LD-MPN protocol was used as the basis for assessing the presence of DD M. tuberculosis in patient sputa.

The LD-MPN and CFU assays, performed as described previously (13) and as outlined in Materials and Methods, both had a lower limit of detection (LLD) of 3 viable M. tuberculosis per milliliter of sputum and upper limits of detection (ULD) of 8.4 × 10^10^ and 3.5 × 10^9^ per ml sputum, respectively. In cases where results were below the LLD, a value of 2 (the highest possible whole number) was recorded for calculation of ratios. Confidence intervals (95%) arising from the 10 replicate dilution series used in the LD-MPN assay were used when scoring DD M. tuberculosis populations as present or absent. We also monitored the time to positivity (TTP, in hours) in duplicate by automated liquid culture (BACTEC MGIT), a semiquantitative measure of viable M. tuberculosis that shows a defined inverse relationship with the CFU per milliliter (14).

**FIG 1:**
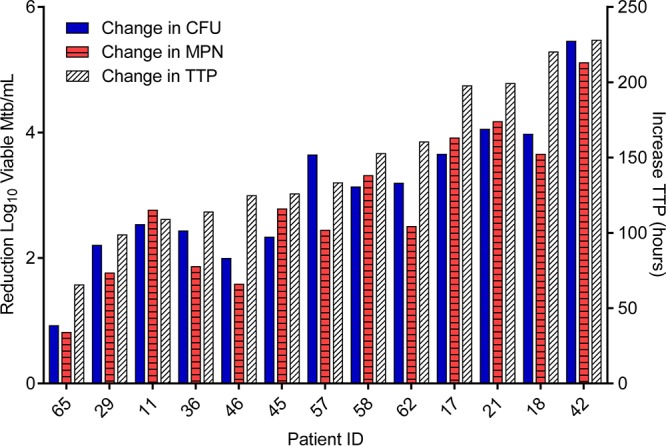
Demonstration of the efficiency of HRZE in reducing the number of viable *M. tuberculosis* (Mtb) over the first 14 days of therapy using 3 quantitative methods: reduction in log_10_ CFU (blue bars), MPN (calculated from LD-MPN assay, red hatched bars), or increase in time to positivity (TTP [in hours], white hatched bars, right *y* axis). Increase in TTP from pretreatment samples to day 12 is plotted for subjects 11 and 21, as a result of contamination of day 14 MGIT cultures for these subjects. Values were below the lower limit of detection (LLD; 3 viable *M. tuberculosis* per ml sputum) in 3 instances at day 14 (subject 11’s MPN and CFU values and subject 42’s CFU value). In these cases, the values were recorded as 2, the highest whole number below the LLD. ID, identification.

Each measure (LD-MPN, CFU, and TTP) detected a substantial decrease in the number of viable M. tuberculosis cells per ml sputum after 14 days of HRZE treatment. This is summarized in Fig. 1, which presents the change in each assay from day 0 (pretreatment) to day 14, not the relative values recorded in each assay. The quantitative values for individual samples and associated 95% confidence intervals (CI) are listed in Table S1 and Fig. S1 and S2 in the supplemental material.

10.1128/mBio.02192-18.3 TABLE S1 MPN of viable *M. tuberculosis* as determined by LD assay and CFU per milliliter of patient sputum for 33 treatment-naive individuals and again after 14 days of HRZE therapy for 13 individuals. All values are calculated per milliliter of original sputum sample and displayed as log_10_ viable *M. tuberculosis* per milliliter of sputum. MPN values are presented with 95% confidence limits. *, values below the lower limit of detection (LLD; 3 viable *M. tuberculosis* per milliliter of sputum) were recorded as 2, the highest whole number below the LLD; 95% confidence intervals are not available for this estimated value. Download Table S1, DOCX file, 0.02 MB. Copyright © 2018 McAulay et al. 2018 McAulay et al. This content is distributed under the terms of the Creative Commons Attribution 4.0 International license.

10.1128/mBio.02192-18.1 FIG S1 CFU (black bars) and most probable number (MPN, gray bars) of viable *M. tuberculosis* as determined by LD assay for 33 treatment-naive individuals (A) and again after 14 days of HRZE therapy for 13 individuals (B). All values were calculated per milliliter of original sputum sample and are displayed as log_10_ viable *M. tuberculosis*; error bars represent 95% confidence limits from MPN calculations. Results below the lower limit of detection (LLD) are marked with a pound symbol. The asterisk marks samples with DD *M. tuberculosis* by the definition that the CFU was below the 95% confidence limit on the MPN value. Download FIG S1, PDF file, 0.3 MB. Copyright © 2018 McAulay et al. 2018 McAulay et al. This content is distributed under the terms of the Creative Commons Attribution 4.0 International license.

10.1128/mBio.02192-18.2 FIG S2 CFU (black bars) and MPN (MPN+CF, gray bars) of viable *M. tuberculosis,* as determined by CF-supplemented LD assay for 33 treatment-naive individuals (A) and again after 14 days of HRZE therapy for 13 individuals (B). All values are calculated per milliliter of original sputum sample and displayed as log_10_ viable *M. tuberculosis*; error bars represent 95% confidence limits from MPN calculations. Samples for which no data are available are marked with a solid diamond. Results below the lower limit of detection are marked with a pound symbol. The asterisk marks samples with DD *M. tuberculosis* by the definition that the CFU was below the 95% confidence limit on the MPN value. Download FIG S2, PDF file, 0.2 MB. Copyright © 2018 McAulay et al. 2018 McAulay et al. This content is distributed under the terms of the Creative Commons Attribution 4.0 International license.

### DD M. tuberculosis measured without CF in sputum from treatment-naive subjects.

**FIG 2:**
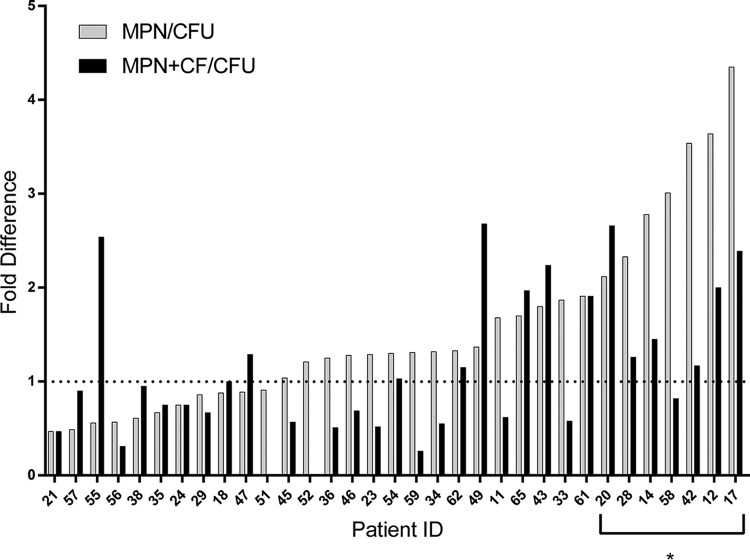
Results (MPN) from limiting dilution (LD) assays and CF-supplemented LD assays (MPN+CF) from sputum from treatment-naive individuals. For each sample, the fold difference is shown for viable bacterial numbers as estimated from the LD-MPN (numerator) assay versus the CFU assay (denominator), where the LD-MPN assay was performed without CF (gray bars) or with CF (solid bars). Data are ordered by size of difference between MPN and CFU. The asterisk marks samples with DD *M. tuberculosis* by the definition that the CFU was below the 95% confidence limit on the MPN value.

For the group of 33 subjects as a whole, the mean outputs from LD-MPN and CFU assays for pretreatment samples were similar (6.5 log_10_ and 6.4 log_10_, respectively), yet the difference was statistically significant by a two-tailed, paired *t* test (*P =* 0.014). For individual subjects, the mean difference between LD-MPN and CFU values was 0.12 log_10_ (range, −0.32 log_10_ to 0.64 log_10_; Fig. 2 and S1a and Table S1), representing an average proportional excess of LD-MPN estimate over CFU of 1.3-fold. For 7 samples (21.2%), the CFU count was lower than the lower bound of the 95% CI for the LD-MPN count. In these 7 samples, the mean difference was 0.48 log_10_ (range, 0.33 to 0.64 log_10_), representing a proportional excess of the LD-MPN estimate over CFU of 3-fold. In only one case did the CFU value exceed the upper bound on the 95% CI for the LD-MPN value. Therefore, 25 samples yielded CFU values within the 95% CI of the respective LD-MPN estimates. When analysis of pretreatment data is restricted to the 13 subjects who were also followed up at day 14, the difference between overall means for MPN and CFU is not significant, though the proportion where CFU were lower than the lower MPN 95% CI remained similar (23.1%).

We detected no statistically significant correlation between subject age or sputum processing time with the presence of DD M. tuberculosis at baseline (one-way analysis of variance [ANOVA]) or of sex or presence of bilateral disease or cavities with the presence of DD M. tuberculosis at baseline (Fisher’s exact test). We also did not detect a statistically significant correlation between bacterial burden in diagnostic samples as assessed by Xpert MTB/RIF (high or medium) or acid-fast bacillus (AFB) smear (+++ or ++) versus Xpert MTB/RIF (low or very low) or AFB smear (+ or −) with the presence of DD M. tuberculosis at baseline (Fisher’s exact test).

### DD M. tuberculosis measured without CF in sputum from subjects at day 14 of standard therapy.

**FIG 3:**
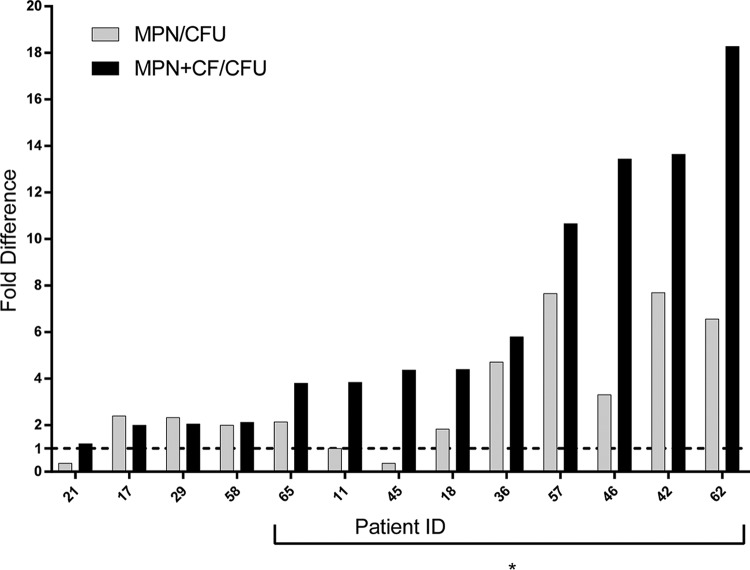
Results (MPN) of limiting dilution (LD) assays and CF-supplemented LD assays (MPN+CF) from sputum samples collected following 14 days of HRZE therapy. For each sample, the fold difference is shown for viable bacterial numbers as estimated from the LD-MPN assay (numerator) versus the CFU assay (denominator), where the LD-MPN assay was performed without CF (gray bars) or with CF (solid bars). Data are ordered by size of difference between MPN+CF and CFU. The asterisk marks samples with DD *M. tuberculosis* by the definition that the CFU was below the 95% confidence limit on the MPN value. Values were below the lower limit of detection (LLD; 3 viable *M. tuberculosis* per ml sputum) in 3 instances (subject 11’s MPN and CFU values and subject 42’s CFU value). In these cases, the values were recorded as 2, the highest whole number below the LLD.

After 14 days of treatment with HRZE, the mean difference between LD-MPN and CFU values was 0.35 log_10_ (range, −0.44 to 0.89 log_10_; *P =* 0.015, two-tailed paired *t* test), representing a proportional excess of LD-MPN over CFU of 2.3-fold. In 8 of the 13 samples (61.5%), the CFU fell below the lower bound of the 95% CI of the LD-MPN estimate, and in these subjects’ specimens, the LD-MPN value exceeded the CFU value by 0.61 log_10_ (range, 0.33 to 0.89 log_10_; Fig. 3 and S1b), representing a proportional excess of LD-MPN over CFU of 4.1-fold. In two cases (15.4%), the CFU value exceeded the upper bound of the 95% CI for the LD-MPN result, with total differences of 0.43 and 0.44 log_10_. The CFU and LD-MPN values were the same in the remaining sample.

We detected no statistically significant correlation between subject age or sputum processing time with DD M. tuberculosis at day 14 (one-way ANOVA) or of sex or presence of bilateral disease or cavities with the presence of DD M. tuberculosis at baseline (Fisher’s exact test). We also did not detect a statistically significant correlation between bacterial burden in diagnostic samples as assessed by Xpert MTB/RIF (high or medium) or AFB smear (+++ or ++) versus Xpert MTB/RIF (low or very low) or AFB smear (+ or −) with the presence of DD M. tuberculosis at day 14 (Fisher’s exact test).

### Contrasting impact of culture filtrate on LD-MPN assays pre- and posttreatment.

For the pretreatment samples, supplementing the LD assay with sterile culture filtrate (CF) harvested from log-phase M. tuberculosis (*n* = 31) yielded a similar mean LD-MPN value as for the standard assay (6.1 log_10_ with CF, 6.2 log_10_ without CF; Table S1 and Fig. S2a), but the difference for individual subjects’ samples was statistically significant (*P =* 0.02, two-tailed, paired *t* test). CFU were lower than the lower bound of the MPN plus CF 95% CI for 6 samples (19.3%). The reduction in estimated viable M. tuberculosis in 58% of the CF-supplemented LD-MPN assays decreased the apparent difference between LD-MPN values and CFU values for pretreatment samples (Fig. 2). When analysis is restricted to the 13 subjects with posttreatment data, the difference between MPN plus CF and CFU is not significant.

Conversely, when applied to sputum samples collected after 14 days of treatment with HRZE, supplementing the LD assay with CF augmented the resulting MPN estimate for 11 of the 13 subjects (84.6%), and all 13 of the supplemented LD assays yielded an MPN greater than the associated value for CFU (Fig. 3 and S2b and Table S1). Here, the mean difference was 0.70 log_10_ (range, 0.08 to 1.44 log_10_), representing a proportional excess of LD-MPN over CFU of 5-fold. The CFU values fell below the lower bound of 95% CI for the supplemented LD-MPN values for 9 subjects (69.2%); the mean difference was 0.90 log_10_ (range, 0.58 to 1.44 log_10_), representing an average proportional excess of DD M. tuberculosis over CFU of 7.9-fold.

## DISCUSSION

The recognition that many environmental and pathogenic bacterial species can enter what was initially dubbed a “viable but nonculturable” (VBNC) state (15, 16) poses interdependent conceptual and practical challenges.

Conceptually, bacteria cannot be assumed to be dead just because they do not begin to replicate soon enough and continue to replicate fast enough on a semisolid nutritive surface to generate biomass visible to the unaided human eye, that is, to be scored as a CFU, before the growth surface desiccates. Diverse stresses can delay the onset or lower the rate of bacterial replication, and diverse injuries can impose the need for repair before a bacterium can give rise to replication-competent progeny. We know of no specific quantifiable metabolic process below a given level of which it is certain that a bacterial cell cannot replicate at some future time. Restated, unless a bacterium has been observed to undergo morphologic disintegration, we have no certainty that it is dead (17).

The practical challenge is to determine how long to wait to assess replicative capacity, and under what conditions of incubation, before attempting to quantitate the number of viable bacteria in a population. By definition, the question is trivial for bacteria under conditions considered standard for that organism in the laboratory, because such conditions are operationally defined as standard when no other conditions have been identified that support faster replication. In contrast, the question is nontrivial for any bacterial population that may have undergone a stress that could impose injury or delay replication, which is to say, bacteria under almost any condition in nature. The question is unanswerable beyond the approximation that any method that gives a higher number is likely to provide a truer estimate, provided that the method is free of artifact, including that the number does not exceed the particle number of morphologically identified bacteria.

In tuberculosis, the intertwined conceptual and practical dilemmas outlined above are clinically relevant and operationally problematic for the following reasons. The course of treatment routinely involves the use of more drugs in combination than for any other bacterial infection and is among the longest in duration for bacterial infections. This makes clinical trials based on relapse-free cure rates exceptionally difficult and puts a premium on choosing the right therapeutic agents, doses, and combinations with which to conduct them. We have no way to measure the total body burden of M. tuberculosis, nor do we have a firm understanding of the variability in clinical outcomes among patients. This gives pivotal importance to the quantification of viable M. tuberculosis in patient sputum over the first 2 weeks of a candidate therapy.

Therefore, we thought it important to ask the following questions: can an independent laboratory applying stringent methodological and statistical criteria confirm that DD M. tuberculosis exists in clinical specimens? If so, are the DD M. tuberculosis cells numerically significant? If so, are the DD M. tuberculosis cells functionally relevant in that their properties differ from the properties of CFU in some way that may plausibly impact the course of disease or the response to treatment? The present results support an affirmative answer to each question, with qualifications.

Application of a modified LD-MPN assay to sputum from a geographically distinct population of subjects with TB confirmed and extended to a distinct patient population the central observation of three earlier studies (9–11) that DD M. tuberculosis is often detectable in the sputum of treatment-naive subjects. The proportion of untreated subjects in whose sputum we detected DD M. tuberculosis by strict statistical criteria (21%) was lower than in the earlier reports (80% [10], 86% [9, 10], and 85% [11]), and the proportionate levels of DD M. tuberculosis were lower, although when DD M. tuberculosis was present, they constituted the majority of the viable M. tuberculosis detected. We do not know if these quantitative differences are statistical, methodological, or biological in origin. Biological variables could include differences in the infecting strains of M. tuberculosis or in genetic, microbiomic, or environmental factors impacting the subjects.

The most striking finding to emerge from this study was that treatment with HRZE for 14 days increased the proportion of subjects with DD M. tuberculosis in their sputum to 69% and increased the proportionate levels of DD M. tuberculosis, so that 7.9-fold more M. tuberculosis was detected by the LD-MPN assay with CF than by CFU, even though the total number of viable M. tuberculosis cells declined in both the CFU and DD M. tuberculosis subpopulations. This is concordant with observations by Mukamolova et al. in sputum from 8 subjects given rifampin alone for 7 to 11 days (10).

It seems likely that one or more of the agents in HRZE increased the proportion of M. tuberculosis in sputum that could be detected by LD and not by CFU. It is not surprising that DD M. tuberculosis may be generated or differentially recovered following anti-TB therapy, as this has been observed in a mouse model with rifampin and with the combination of isoniazid and pyrazinamide (18), as well as in models *in vitro* following exposure to rifampin, isoniazid, or ethambutol (19). In a study that controlled for potential antibiotic carryover to agar plates, DD M. tuberculosis populations were observed *in vitro* only after sequential starvation of M. tuberculosis and exposure to rifampin and not with starvation followed by exposure to isoniazid, ethambutol, or pyrazinamide (13).

Whether the impact of HRZE on the increased proportion of DD M. tuberculosis among residual viable M. tuberculosis was attributable to rifampin alone, to another drug, or to a combination of drugs, we conclude that a drug regimen that is demonstrably effective in lowering the burden of CFU can at the same time leave the majority of the surviving M. tuberculosis in a form that is not detectable as CFU. While some such bacteria may be on their way to death, at least some of the M. tuberculosis cells that are not CFU recover proliferative potential following LD. At that point, they presumably have disease-causing potential as well. It seems prudent to assume that DD M. tuberculosis as found in sputum is also present in lesions from which the sputum arises and in aerosols that arise from the same sources. If so, it is a significant concern that DD M. tuberculosis remaining in the patient and transiting by aerosol to others may have disease-causing potential.

A recent study detected M. tuberculosis mRNA in culture-negative sputum from 35% of patients after 6 months of treatment (20). The authors concluded, “we suggest that viable MTB…often persist even after clinically curative treatment” (20). M. tuberculosis strains that can be considered viable only by a criterion other than standard culture methods are, by definition, DD M. tuberculosis, whether or not they are demonstrable following an LD assay such as that used here. In contrast, another recent study based on an estimation of M. tuberculosis DNA in sputum samples suggested that the apparent initial efficacy of therapy argues against the relevance of DD M. tuberculosis (21). The results of that work are difficult to interpret without associated culture-based quantification, given the variability in the presence of DD M. tuberculosis found before treatment in our study. Additionally, it is not clear whether chemical modifications or reparable breaks in DNA could lead to the underdetection of M. tuberculosis by the Xpert MTB/RIF assay. It also is unknown if all forms of DD M. tuberculosis are phenotypically tolerant to current medications, but our results suggest that at least one DD M. tuberculosis subpopulation persists after 2 weeks of standard therapy, and this can be studied further for clinical relevance.

The role of CF in LD-MPN assays remains biologically unexplained and methodologically ambiguous. In some studies, nonreplicating M. tuberculosis begins to replicate following exposure to resuscitation-promoting factors (Rpfs) (22, 23), a family of proteins not restricted to mycobacteria (24), some of which have peptidoglycan hydrolase activity (25–28). CF is a source of Rpfs, along with other factors reported to impact M. tuberculosis growth (29–31). The addition of CF to culture medium has improved M. tuberculosis yield *in vitro* and *ex vivo* to various degrees (9, 10, 18, 19, 32, 33), although this has also been observed with CF prepared from M. tuberculosis in which all *rpf* genes have been deleted (9). In the largest reported study of DD M. tuberculosis in sputum samples, CF sometimes enhanced and sometimes suppressed the values of LD-MPN assays; neither effect could be attributed to Rpfs (9). In the present study, the effect of CF was variable, but on average, CF suppressed LD-MPN numbers for sputa obtained before treatment and enhanced LD-MPN numbers for sputa obtained after treatment.

Clinically, the use of standard culture methods to monitor sputum culture conversion from growth of M. tuberculosis to no growth of M. tuberculosis after 2 months of treatment performs poorly in predicting outcomes (34). Indeed, an attempt to use 2-month culture conversion as a criterion to shorten standard therapy to 4 months resulted in more relapses (35). Furthermore, studies attempting to shorten therapy by including a fluoroquinolone were inferior to 6-month control regimens, despite phase II trial data demonstrating increased 2-month culture conversion with the use of fluoroquinolones (36–39). Our results suggest that DD M. tuberculosis populations might contribute to the failure of traditional sputum culture methods as surrogates for clinical outcome following treatment. Understanding this phenomenon could be critically important as new regimens are investigated, including those testing higher doses of rifampin (40).

These considerations lead us to suggest that the LD assay be performed both with and without CF, along with CFU and/or MGIT assays. The resulting burden on a clinical microbiology laboratory is considerable, underscoring the imperative to learn enough about the molecular features of DD M. tuberculosis to devise a simpler assay. If assessment of DD M. tuberculosis levels could be incorporated more widely into clinical trials, it could be possible to test the hypothesis that their elimination will mark a cure. Furthermore, the heterogeneity among patients’ cultures both before and during treatment in our study suggests there is potential or even need for individualization of treatment. This could allow regimens to be shortened for subjects who respond more quickly than others to currently approved regimens, and perhaps for all subjects who receive regimens containing drugs that overcome the phenotypic tolerance of nonreplicating M. tuberculosis. Studies are planned to follow changes in DD M. tuberculosis over longer times of treatment.

## MATERIALS AND METHODS

### Study population and specimen collection.

Overnight pooled sputum was collected from subjects prior to the initiation of anti-TB therapy (*n* = 33) and after 14 days therapy with standard four-drug therapy (isoniazid, rifampin, pyrazinamide, and ethambutol [HRZE]; *n* = 13). As will be reported elsewhere, at the conclusion of the present study, all subjects who had not yet received HRZE began HRZE, and all those who had received HRZE continued the regimen. Subjects were instructed to collect all sputum produced between the hours of 17:00 and 9:00 in a single container, which was kept in a prechilled cool box containing ice packs. Study subjects were housed at the same site as the GHESKIO IMIS Mycobacteriology Laboratory, minimizing specimen transfer time. Upon receipt in the laboratory, sputum samples were immediately refrigerated and processed within 48 h, with the exception of one sample, which was stored at 2 to 8°C for 4 days prior to decontamination.

### Sputum decontamination.

Sputum was transferred to 50-ml screw-top centrifuge tubes (≤30 ml per tube) and vortexed for 5 min to promote homogenization. Homogenized sputum (5 ml) was decontaminated with 5 ml NALC-NaOH (3% sodium hydroxide, 0.5 to 0.6% *N*-acetyl-l-cysteine, 1.47% sodium citrate) by vortexing at 5-min intervals for 20 min. For batch preparations of NALC-NaOH, NALC was added to 0.5%. When prepared for individual samples, one 30-mg tablet was used per specimen (0.6%; Alpha-Tec Systems, Vancouver, WA). Neutralization was achieved by adding 35 ml of 67 mM phosphate (Na-K) buffer (pH 6.8), followed by centrifugation at 2,192 × *g* for 15 min at 16°C. Pellets were resuspended in 3 ml phosphate buffer (subjects 11 to 21) or 7H9 medium (subjects 23 to 65). Decontaminated sputum was used immediately in LD assays (*n* = 21) or after storage at 2 to 8°C for less than 24 h (*n* = 17) or less than 72 h (*n* = 7); one sample was stored at 2 to 8°C for 18 days.

### Preparation of CF.

Bacteriologic media and oleic acid-albumin-dextrose-catalase (OADC) were purchased from Becton Dickinson (BD, Franklin Lakes, NJ). Wild-type Mycobacterium tuberculosis H37Rv (ATCC 27294) was kindly provided by Bavesh Kana (University of Witwatersrand) and used to prepare CF. A frozen stock was inoculated into 8 ml Middlebrook 7H9 broth with 0.2% glycerol, 10% OADC, and 0.02% tyloxapol (hereafter called 7H9), incubated under standing conditions at 37°C in vented tissue culture flasks for 5 to 7 days, and then passaged to 30 ml and incubated until exponential phase (optical density at 600 nm [OD_600_], 0.4 to 0.8). Cells were pelleted by centrifugation at 2,192 × *g* for 15 min at 16°C, and the supernatant was passed through a 0.2-µm filter to remove any residual cells.

### CFU assays.

The CFU per milliliter were determined on Middlebrook 7H10 or 7H11 agar supplemented with OADC in partially sealed plastic bags at 37°C. For each CFU assay, 16 µl of each of 5 replicates from independent dilution series for the LD assay described below were pooled in a corresponding well in a new 96-well plate. The pools were mixed by pipette, and 10 µl from each of dilutions 1 to 6 was spread on sections of 7H10 triplates. The same procedure was performed separately with another 5-replicate independent dilution series. Additionally, 300 µl of decontaminated sputum was diluted 1:1 with 300 µl 7H9 that was supplemented with 4% (vol/vol) of an antimicrobial cocktail (BBL PANTA [polymyxin B, amphotericin B, nalidixic acid, trimethoprim, and azlocillin]; BD) and spread on two 7H11 plates. Plates were read after 3, 5, 7, and 9 weeks; dilutions generating 10 to 150 CFU per triplate section were used to calculate the CFU per milliliter. Individual assays were terminated after 5, 7, or 9 weeks if no change in colony count was observed from the previous reading. The reported CFU is the average of the two sets of pooled dilution series.

### MGIT assay.

Automated liquid culture was performed using a BACTEC 960 instrument (Becton Dickinson [BD], Franklin Lakes, NJ) and mycobacterial growth indicator tubes (MGITs), according to the manufacturers’ instructions.

### LD assay.

CF was mixed at a 1:1 ratio with 7H9, and PANTA was added to a final concentration of 4% (vol/vol); this mixture (7H9+CF) was then used immediately where indicated. The LD assay was performed as described previously (13), with minor modifications. Briefly, for each assay, all wells of two 96-well flat-bottomed microtiter plates were filled with 216 µl of 7H9 plus CF, and all wells of another two plates were filled with 216 µl of 7H9 supplemented with 4% (vol/vol) PANTA but without CF. Decontaminated sputum was vortexed to disperse clumps before transferring 24 µl to wells B to F in column 2, initiating 10 independent dilution series for both 7H9 and 7H9+CF. Wells were mixed by pipetting up and down in a circular motion 10 times using filtered tips, and then 24 µl was transferred to rows B to F in column 3. These 10-fold dilutions were repeated across to column 11, changing tips with each dilution. Plates were incubated at 37°C in partially sealed plastic bags and read at the same intervals as the CFU plates. Individual assays were terminated at 5, 7, or 9 weeks if results had not changed over the past 2 weeks. The presence or absence of growth in each well was recorded in a binary fashion at each time point, and results passing the quality control measures listed below were used in an MPN calculation (12) to determine the number of viable organisms per milliliter in the original sputum sample. These steps have been detailed in a written protocol and illustrated in a published video (13).

### Quality control.

Positive MGIT cultures were inoculated on blood agar, and those exhibiting evidence of contamination were excluded from TTP analysis. For each batch of CF, the cell pellet and CF (100 µl each) were inoculated on blood agar and incubated at 37°C for 7 days to monitor for non-M. tuberculosis contamination, and the CF was monitored for mycobacterial contamination via MGIT culture. After each final LD-MPN read, terminal wells showing growth were monitored for non-M. tuberculosis growth by inoculating on blood agar and incubating at 37°C for 7 days.

### Data analysis.

We calculated 95% confidence intervals for MPN values from the results of 10 independent dilution series per sample (12). All quantitative results are presented as log_10_ values per milliliter of sputum. LD-MPN assays are compared to CFU assays by subtracting the log_10_ value for the CFU assays (the subtrahend) from the log_10_ value for the LD-MPN assays (the minuend) and presenting the difference. The antilog of the difference represents the ratio between the LD-MPN assay result (numerator) and the CFU assay result (denominator). Similarly, LD-MPN assays performed in the presence of 50% freshly prepared culture filtrate (CF) from an exponentially replicating M. tuberculosis culture are compared to LD-MPN assays performed without CF by subtracting the log_10_ value of the assays performed without CF from the log_10_ value of the assays performed with CF. LD-MPN assays were performed with 10 independent dilution series per sample and are presented with 95% confidence limits (95% CI) derived from the variation among these replicates. CFU counts were made by plating aliquots from each of two dilution series made by pooling aliquots from five of the independent dilution series used for the LD-MPN assay, generating 2 CFU values for LD-MPN and 2 for LD-MPN+CF; the reported results for CFU are the average of the two resulting values under each set of conditions. DD M. tuberculosis populations were recorded as present when their number was computed from a CFU value that was less than the lower 95% confidence interval of the LD-MPN value. The few instances in which the CFU value was higher than the upper 95% confidence interval of the LD-MPN value are considered technical failures and illustrate that both assays are imperfect. We used a two-tailed paired *t* test to compare means of two continuous features (MPN versus CFU) from the same patients. ANOVA was used to compare means of continuous features of independent groups (age and sputum processing time). Fisher’s exact test was used to assess associations between two categorical features (Xpert MTB/RIF high or low, AFB score high or low). Statistical analyses were performed using GraphPad Prism version 7.04 for Windows (GraphPad Software, La Jolla, CA, USA).
