# Supplementary material for: Differentially Detectable Mycobacterium tuberculosis Cells in Sputum from Treatment-Naive Subjects in Haiti and Their Proportionate Increase after Initiation of Treatment
Source: mBio. 2018 Nov 20;9(6):e02192-18. doi: 10.1128/mBio.02192-18 (PMC6247085; doi:10.1128/mBio.02192-18)
Supplement: TABLE S1 [file mbo006184173st1.docx]

Table S1

| **Patient Number** | **Sample Number** | **Treatment Status** | **Storage prior to decontamination (days)** | **Storage prior to LD-MPN assay (days)** | **Log10 CFU** | **Log10 MPN (95% CI)** | **Log10 MPN+CF (95% CI** |
| --- | --- | --- | --- | --- | --- | --- | --- |
| 3041011 | 1 | Pre-treatment | 4 | 0 | 2.54 | 2.77 (3.08, 2.47) | 2.34 (2.7, 1.96) |
| 3041012 | 2 | Pre-treatment | 1 | 1 | 6.59 | 7.15 (7.5, 6.79) | 6.89 (7.15, 6.59) |
| 3041014 | 3 | Pre-treatment | 0 | 2 | 6.06 | 6.51 (6.85, 6.17) | 6.23 (6.59, 5.89) |
| 3041017 | 4 | Pre-treatment | 1 | 0 | 6.51 | 7.15 (7.5, 6.79) | 6.89 (7.19, 6.59) |
| 3041018 | 5 | Pre-treatment | 0 | 0 | 6.23 | 6.17 (6.46, 5.89) | 6.23 (6.59, 5.89) |
| 3041020 | 6 | Pre-treatment | 1 | 1 | 6.34 | 6.67 (6.96, 6.36) | 6.77 (7.08, 6.47) |
| 3041021 | 7 | Pre-treatment | 0 | 1 | 6.55 | 6.23 (6.59, 5.89) | 6.23 (6.59, 5.89) |
| 3041023 | 8 | Pre-treatment | 0 | 0 | 7.72 | 7.83 (8.15, 7.52) | 7.44 (7.79, 7.08) |
| 3041024 | 9 | Pre-treatment | 0 | 0 | 7.46 | 7.34 (7.7, 6.96) | 7.34 (7.7, 6.96) |
| 3041028 | 10 | Pre-treatment | 1 | 1 | 6.86 | 7.23 (7.59, 6.89) | 6.96 (7.24, 6.65) |
| 3041029 | 11 | Pre-treatment | 1 | 1 | 6.61 | 6.54 (6.89, 6.21) | 6.44 (6.79, 6.08) |
| 3041033 | 12 | Pre-treatment | 2 | 0 | 7.06 | 7.34 (7.7, 6.96) | 6.83 (7.15, 6.52) |
| 3041034 | 13 | Pre-treatment | 0 | 0 | 7.22 | 7.34 (7.7, 6.96) | 6.96 (7.24, 6.65) |
| 3041035 | 14 | Pre-treatment | 0 | 0 | 6.89 | 6.71 (7.02, 6.4) | 6.76 (7.1, 6.44) |
| 3041036 | 15 | Pre-treatment | 0 | 0 | 6.73 | 6.83 (7.12, 6.54) | 6.44 (6.79, 6.08) |
| 3041038 | 16 | Pre-treatment | 0 | 1 | 6.36 | 6.15 (6.5, 5.79) | 6.34 (6.7, 5.96) |
| 3041042 | 17 | Pre-treatment | 0 | 0 | 5.76 | 6.31 (6.66, 5.96) | 5.83 (6.15, 5.52) |
| 3041043 | 18 | Pre-treatment | 0 | 0 | 4.16 | 4.41 (4.76, 4.05) | 4.51 (4.85, 4.17) |
| 3041045 | 19 | Pre-treatment | 0 | 1 | 6.21 | 6.23 (6.59, 5.89) | 5.96 (6.24, 5.65) |
| 3041046 | 20 | Pre-treatment | 0 | 2 | 6.31 | 6.41 (6.76, 6.05) | 6.15 (6.5, 5.79) |
| 3041047 | 21 | Pre-treatment | 2 | 0 | 6.66 | 6.61 (6.92, 6.29) | 6.77 (7.08, 6.47) |
| 3041049 | 22 | Pre-treatment | 1 | 1 | 6.01 | 6.15 (6.5, 5.79) | 6.44 (6.79, 6.08) |
| 3041051 | 23 | Pre-treatment | 2 | 1 | 5.38 | 5.34 (5.7, 4.96) | No data |
| 3041052 | 24 | Pre-treatment | 2 | 1 | 6.06 | 6.15 (6.5, 5.79) | No data |
| 3041054 | 25 | Pre-treatment | 2 | 2 | 6.32 | 6.44 (6.79, 6.08) | 6.34 (6.7, 5.96) |
| 3041055 | 26 | Pre-treatment | 0 | 1 | 6.82 | 6.57 (6.89, 6.26) | 7.23 (7.59, 6.89) |
| 3041056 | 27 | Pre-treatment | 2 | 1 | 7.39 | 7.15 (7.5, 6.79) | 6.89 (7.19, 6.59) |
| 3041057 | 28 | Pre-treatment | 0 | 0 | 7.27 | 6.96 (7.24, 6.65) | 7.23 (7.59, 6.89) |
| 3041058 | 29 | Pre-treatment | 1 | 2 | 6.86 | 7.34 (7.7, 6.96) | 6.77 (7.08, 6.47) |
| 3041059 | 30 | Pre-treatment | 1 | 2 | 6.61 | 6.73 (7.02, 6.44) | 6.02 (6.31, 5.71) |
| 3041061 | 31 | Pre-treatment | 2 | 1 | 6.37 | 6.65 (6.99, 6.32) | 6.65 (6.99, 6.32) |
| 3041062 | 32 | Pre-treatment | 2 | 1 | 6.59 | 6.71 (7.02, 6.4) | 6.65 (6.99, 6.32) |
| 3041065 | 33 | Pre-treatment | 0 | 0 | 6.42 | 6.65 (6.99, 6.32) | 6.71 (7.02, 6.4) |
| 3041011 | 34 | Day 14 RHEZ | 0 | 3 | 0.3* | 0.3* | 0.89 (1.51, 0.28) |
| 3041017 | 35 | Day 14 RHEZ | 0 | 2 | 2.85 | 3.23 (3.59, 2.89) | 3.15 (3.5, 2.79) |
| 3041018 | 36 | Day 14 RHEZ | 0 | 0 | 2.24 | 2.51 (2.85, 2.17) | 2.89 (3.19, 2.59) |
| 3041021 | 37 | Day 14 RHEZ | 0 | 0 | 2.49 | 2.05 (2.4, 1.71) | 2.57 (2.89, 2.26) |
| 3041029 | 38 | Day 14 RHEZ | 0 | 0 | 4.40 | 4.77 (5.08, 4.47) | 4.71 (5.02, 4.4) |
| 3041036 | 39 | Day 14 RHEZ | 0 | 0 | 4.29 | 4.96 (5.24, 4.65) | 5.05 (5.4, 4.71) |
| 3041042 | 40 | Day 14 RHEZ | 0 | 0 | 0.3* | 1.19 (1.63, 0.75) | 1.44 (1.8, 1.08) |
| 3041045 | 41 | Day 14 RHEZ | 0 | 18 | 3.87 | 3.44 (3.79, 3.08) | 4.51 (4.85, 4.17) |
| 3041046 | 42 | Day 14 RHEZ | 1 | 1 | 4.31 | 4.83 (5.15, 4.52) | 5.44 (5.79, 5.08) |
| 3041057 | 43 | Day 14 RHEZ | 0 | 0 | 3.62 | 4.51 (4.85, 4.17) | 4.65 (4.99, 4.32) |
| 3041058 | 44 | Day 14 RHEZ | 1 | 1 | 3.72 | 4.02 (4.31, 3.71) | 4.05 (4.4, 3.71) |
| 3041062 | 45 | Day 14 RHEZ | 2 | 1 | 3.39 | 4.21 (4.57, 3.85) | 4.65 (4.99, 4.32) |
| 3041065 | 46 | Day 14 RHEZ | 2 | 0 | 5.49 | 5.83 (6.15, 5.52) | 6.08 (6.36, 5.77) |

**Table S1**. Most probable number (MPN) of viable Mtb as determined by LD assay and CFU/mL of patient sputum for 33 treatment-naïve individuals and again after 14 days of HRZE therapy for 13 individuals. All values are calculated per mL original sputum sample and displayed as log_10_ viable Mtb/mL sputum. MPN values are presented with 95% confidence limits.

*Values were below the lower limit of detection (LLD; 3 viable Mtb per mL sputum) were recorded as 2, the highest whole number below the LLD (0.3 log_10_); 95% confidence intervals are not available for this estimated value.
