# Supplementary figures and images for: Differentially Detectable Mycobacterium tuberculosis Cells in Sputum from Treatment-Naive Subjects in Haiti and Their Proportionate Increase after Initiation of Treatment
Source: mBio. 2018 Nov 20;9(6):e02192-18. doi: 10.1128/mBio.02192-18 (PMC6247085; doi:10.1128/mBio.02192-18)

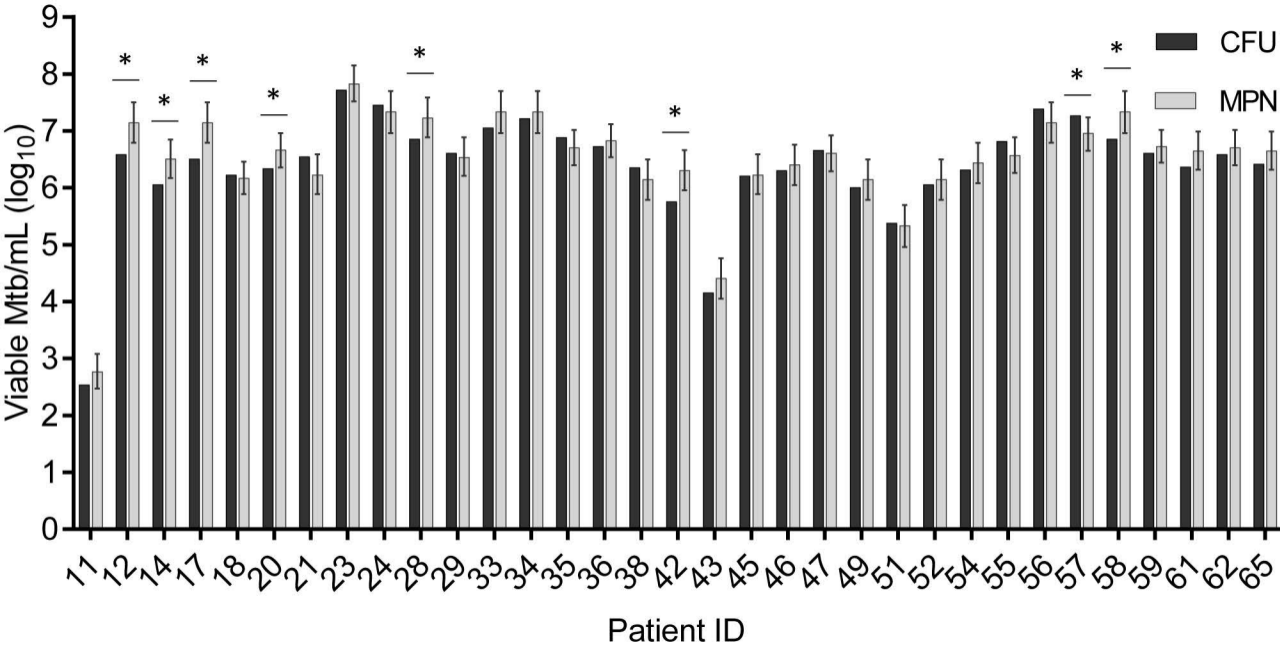

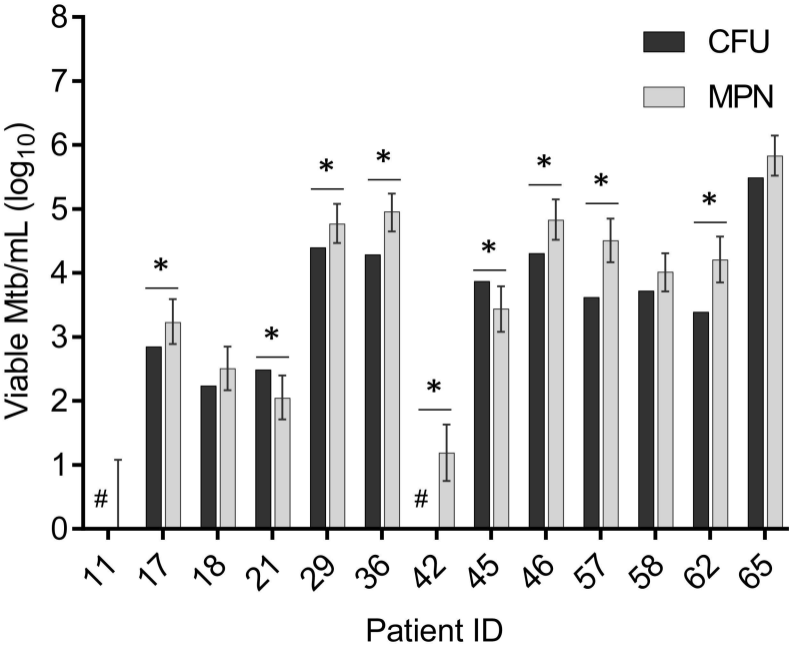

Supplement: FIG S1 [file mbo006184173sf1.pdf]

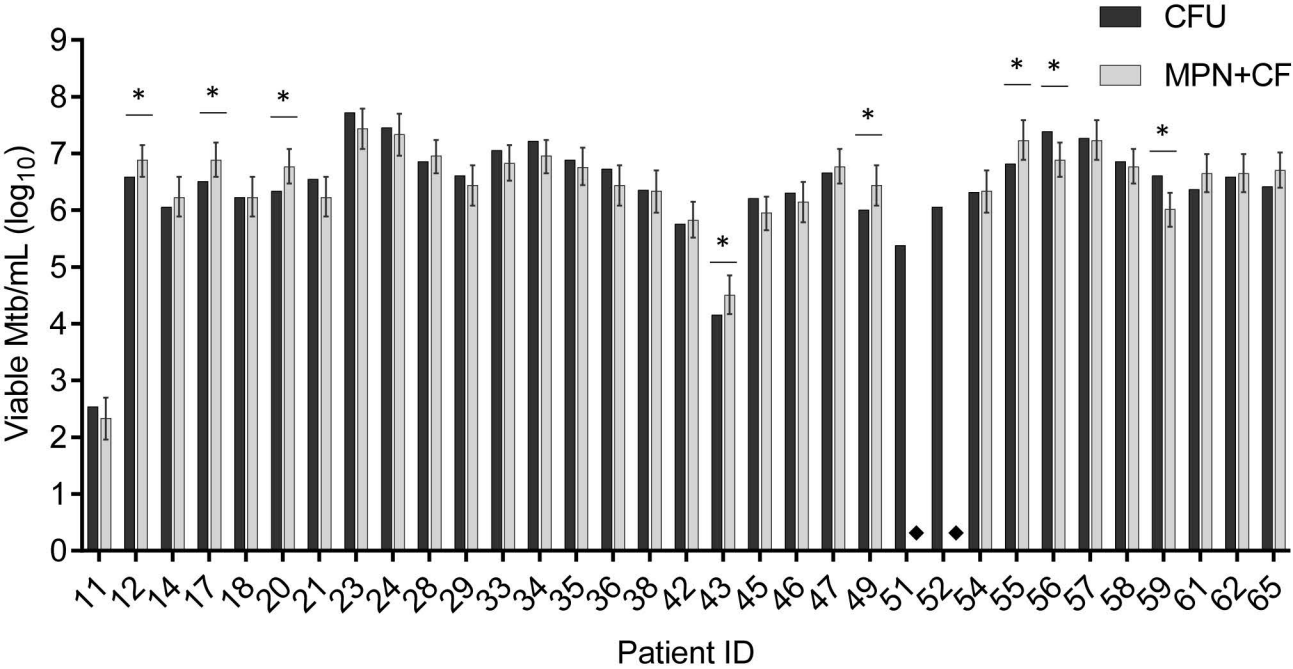

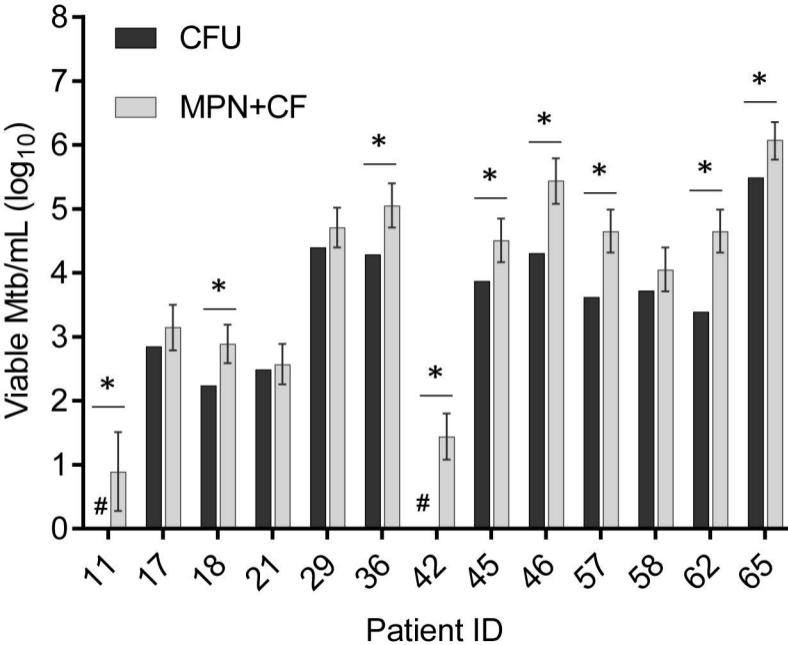

Supplement: FIG S2 [file mbo006184173sf2.pdf]
